# Editorial: Metals in cancer: from intracellular signaling to therapy

**DOI:** 10.3389/fonc.2024.1495825

**Published:** 2024-10-10

**Authors:** Katia Aquilano, Giuseppe Filomeni, Raffaella Faraonio, Anastasia De Luca

**Affiliations:** ^1^ Department of Biology, University of Rome Tor Vergata, Rome, Italy; ^2^ Redox Biology Research Group, Danish Cancer Institute, Copenhagen, Denmark; ^3^ Department of Molecular Medicine and Medical Biotechnology, University of Naples Federico II, Naples, Italy

**Keywords:** trace metals, heavy metals, cancer, neurodegeneration, cell signaling

The role of metals in biology has evolved far beyond their traditional function as enzyme cofactors or inhibitors. In 2002, Dixon introduced the term “ferroptosis” to describe an iron-dependent and apoptosis-independent type of cell death that plays a significant role in the elimination of cancer cells (1). Twelve years later, the term “cuproptosis” was coined to define a mitochondrial copper-dependent mechanism of cell death that holds the potential to counteract the spread of cancer (2). Recently, the intricate interplay between these two metal-dependent cell death pathways has been further explored (3, 4). Bian et al. provided a comprehensive review of the role of intracellular copper bioavailability in cancer onset and development, emphasizing the interconnection between cuproptosis and ferroptosis, and discussing the ongoing clinical trials of copper-based chemotherapeutic strategies. The authors considered the evolution of the clinical use of copper chelators and ionophores (molecules capable of increasing intracellular copper concentration), highlighting one of the main limitations of these therapeutic approaches. In fact, the systemic administration of these molecules is likely to result in adverse side effects. For this reason, current research is focusing on identifying drug delivery systems specifically targeting the tumor mass.

Dysregulation of zinc (5), selenium (6), and magnesium (7) homeostasis has also been implicated in cancer onset and progression. Similarly, it is well established that an intracellular excess of heavy metals (e.g., lead, mercury, and chromium) can induce tumorigenesis. Heavy metal intoxication is also implicated in the development of several neurological conditions and in the activation of cellular senescence, as observed in these neurological pathologies (8). Pamphlett and Bishop, through elemental biomapping of various tissues, were able to measure a range of heavy metals including inorganic mercury, silver, bismuth, cadmium, lead, and nickel in several tissue samples from the kidney, pancreas, thyroid, nervous system, breast and anterior pituitary. Their study of 170 autopsies from patients with hypertension, pancreatic cancer, breast cancer, gliomas, multiple sclerosis, neurodegenerative disorders (*i.e.*, Parkinson’s disease, age-related macular degeneration), and from aged patients, reveals that aging cells that accumulate heavy metals are more likely to undergo malignant transformation. In particular, they find an age-dependent accumulation of mercury in cell populations such as renal tubule cells in the kidney, b-cells in the pancreas, and follicular epithelium in the thyroid. In the nervous system, mercury and silver accumulation is mainly observed in the locus ceruleus and in the hypophysis. Additionally, they observed increased concentrations of mercury in pancreatic and breast cancer tissues, reinforcing the notion that toxic metal accumulation during aging contributes to tumorigenesis, and suggesting that reducing environmental contamination by heavy metals could mitigate cancer incidence.

These findings have strongly promoted and supported the synthesis and development of metal-based chemotherapeutic strategies to exploit their antitumor properties. In particular, several copper- and iron-based compounds have been proposed to increase the intracellular bioavailability of these trace metals in cancer cells, thereby triggering cuproptosis or ferroptosis. In this regard, platinum-based compounds (*i.e.*, carboplatin, oxaliplatin, and cisplatin) remain among the most commonly used drugs for the treatment of various cancers, such as lung, ovarian, cervical, testicular, bladder, and colorectal cancers. However, over the last decade, researchers have also focused on identifying nature-derived bioactive compounds capable of inhibiting cancer through the induction of non-canonical cell death signaling pathways (9) or functioning as adjuvants to standard cancer therapies. In their original study, Yang et al. demonstrated the synergistic activity of the lignan Schisandrin C (SC) and cisplatinum in a mouse model of breast and colon cancer. This polyphenol acts by increasing cisplatin-induced activation of type I interferon (IFN) signaling, leading to improved antitumor immune responses. Specifically, they provided evidence that SC increases the type I IFN cascade mediated by the cyclic GMP-AMP synthase (cGAS)-stimulator of interferon genes (STING) pathway of innate immunity. Additionally, SC enhances the recruitment of T lymphocytes and NK cells to the tumor site. Simultaneous administration of SC and cisplatin not only significantly improves overall survival (OS) compared to cisplatin alone but also improves cisplatin tolerability, limiting weight loss in tumor-bearing mice.

In an alternative approach, Wang et al. proposed a cisplatin-independent treatment for non-oncogene-addicted (NOA) advanced non-small cell lung cancer (NSCLC). Their multicenter retrospective study of 87 patients who were unresponsive to first-line platinum-based doublet chemotherapy, demonstrated that the treatment with the S-1 molecule, composed of tegafur (FT), gimeracil (CDHP), and oteracil potassium (OXO), significantly improves progression-free survival (PFS) and overall survival (OS), with better tolerability in NSCLC patients compared to platinum-based regimens, thus supporting its potential use as a second-line of intervention in cancer treatment.

Overall, the studies featured in this Research Topic underscore the dual role of metals in cancer both as drivers of tumorigenesis and as therapeutic agents. Understanding the intricate balance of metal homeostasis and exploiting metal-dependent cell death pathways offer new potential avenues for cancer treatment. Future research is required to refine these strategies, to minimize the risks posed by toxic metal accumulation while leveraging their potential for targeted therapies.
